# Herb-Partitioned Moxibustion Improves Crohn's Disease-Associated Intestinal Fibrosis by Suppressing the RhoA/ROCK1/MLC Pathway

**DOI:** 10.1155/2021/2247953

**Published:** 2021-11-17

**Authors:** Min Zhao, Zhijun Weng, Yan Huang, Handan Zheng, Dong Han, Jiacheng Shen, Rong Huang, Huirong Liu, Luyi Wu

**Affiliations:** ^1^Key Laboratory of Acupuncture and Immunological Effects, Shanghai University of Traditional Chinese Medicine, Shanghai 200030, China; ^2^Shanghai Research Institute of Acupuncture and Meridian, Shanghai 200030, China

## Abstract

**Background and Aims:**

Intestinal fibrosis is one of the severe and common complications of Crohn's disease (CD), but the etiology and pathogenesis remain uncertain. The study intended to examine whether the effect of herb-partitioned moxibustion on rats with CD-associated intestinal fibrosis is associated with the RhoA/ROCK1/MLC pathway.

**Methods:**

All experimental rats were randomly allocated into the normal control group (NC), model control group (MC), and herb-partitioned moxibustion group (HPM). Intestinal fibrosis was established in rats with CD by repeated rectal administrations of 2,4,6-trinitrobenzenesulfonic acid (TNBS). Herb-partitioned moxibustion was applied at the Qihai (CV6) and Tianshu (ST25) acupoints once daily for 10 days in the HPM group. In this study, histological changes were examined by hematoxylin and eosin (HE) staining; then, Masson's trichrome staining was used to assess the degree of fibrosis in each group. Experimental methods of immunohistochemistry, western blotting, and real-time PCR were applied to detect the levels of *α*-SMA, collagen III, RhoA, ROCK1, and p-MLC. Moreover, the double immunofluorescent staining for the colocalization of both *α*-SMA and ROCK1 was performed.

**Results:**

Contrasted with the normal controls, the collagen deposition and fibrosis scores were increased in colonic tissue of model rats, and HPM decreased the collagen deposition and fibrosis scores. The protein of *α*-SMA and collagen III in the MC group exceeds that of the NC group; HPM decreased the expression of *α*-SMA and collagen III in rats with intestinal fibrosis. Similarly, the expression of RhoA, ROCK1, and p-MLC in model rats was obviously increased compared with normal controls; the expression of RhoA, ROCK1, and p-MLC was decreased after HPM. The coexpression of *α*-SMA and ROCK1 in rats with intestinal fibrosis was higher than normal rats.

**Conclusion:**

HPM improves CD-associated intestinal fibrosis by suppressing the RhoA/ROCK1/MLC pathway.

## 1. Introduction

Intestinal fibrosis is one of the severe and common complications of Crohn's disease (CD), which can invade almost all parts of the digestive tract and involve the entire intestinal wall, with abdominal pain, constipation, lumen narrowing, and even intestinal obstruction or intestinal perforation [1]. Clinically, over half of the CD patients suffer from surgical intervention due to intestinal fibrosis, but the restenosis rate is still as high as 70%, and can even lead to short bowel syndrome [2–4], so the disease causes a heavy burden to both families and society. Intestinal fibrosis is the result of repeated injury and excess repair; however, the pathological mechanism is still unclear and no specific antifibrotic therapies are currently available [5]. Therefore, it is urgent to explore its pathogenesis and new therapy.

Intestinal fibrosis has the distinction of fibroblast proliferation and the deposition of extracellular matrix (ECM). Excessive intestinal repair mainly involves the migration of fibroblasts and cytoskeletal remodeling [6]. As a member of the Rho family protein, RhoA is a transducer in cell signal transduction. Rho-associated coiled-coil containing protein kinase 1 (ROCK1) is the main downstream signaling molecule of RhoA. The RhoA/ROCK1 pathway plays a vital role in cytoskeletal remodeling and regulates fundamental cellular processes, including cell motility, contraction, adhesion, and proliferation [7, 8]. The RhoA/ROCK1 pathway participates in the activation of myofibroblasts in primary cultures of human intestinal myofibroblasts [9, 10]. A preclinical study showed that ROCK was activated in intestinal fibroblasts, epithelial cells, endothelial cells, and muscle cells in patients with CD-associated intestinal fibrosis [11]. As the downstream effector protein of RhoA, MCL is phosphorylated by the activation of RhoA/Rho kinase. MLC activation is related to disease activity in inflammatory bowel disease patients [12]. RhoA/ROCK1 pathway inhibitors have also been shown to reverse the degree of intestinal fibrosis [13, 14]. Therefore, we hypothesize that the RhoA/ROCK1/MLC pathway may take part in the pathological process of intestinal fibrosis.

Currently, the common treatment about CD-associated intestinal fibrosis only focuses on anti-inflammatory rather than antifibrosis; the use of biological agents does not significantly alleviate this disease [15]. However, it has been shown that moxibustion has the effect of antifibrosis [16, 17]. Our team has found that HPM can significantly inhibit epithelial mesenchymal transition (EMT), reduce collagen deposition, and improve intestinal fibrosis in CD rats [18, 19]. In the research, 2,4,6-trinitrobenzenesulfonic acid (TNBS) was used to establish the model of CD-associated intestinal fibrosis. The degree of intestinal fibrosis was examined by histopathology; the level of *α*-SMA, collagen III, ROCK1, RhoA, and p-MLC was measured to elucidate the mechanism through immunohistochemistry, western blotting, and real-time PCR, by which HPM affects CD-associated intestinal fibrosis.

## 2. Materials and Methods

### 2.1. Experiment Animals

All the animals in this study, twenty-four male SPF-grade Sprague Dawley rats (200 ± 20 g), were provided by the Experimental Animal Center at the Yueyang Hospital of Integrated Traditional Chinese and Western Medicine, Shanghai University of Traditional Chinese Medicine. The animals were housed under standard conditions at 24 ± 1°C and a 12 h light and shade cycle with 50–70% ambient humidity. Rats had free access to water and feed themselves. All the experiments in this study were permitted by the ethics committee of Yueyang Hospital of Integrated Traditional Chinese and Western Medicine, Shanghai University of Traditional Chinese Medicine (no. YYLAC-2019-045-1).

### 2.2. Groupings and Model Establishment

All rats were randomly divided into the normal control (NC), model control (MC), and HPM group, which were deprived of food for 24 hours before treatment. There are eight rats in each group. The NC group was administered enemas with normal saline, and the other groups were administered weekly enemas with TNBS for 6 weeks to establish the experimental model [20]. The enemas consist of double 5% TNBS mixed with single 50% ethanol. The rats accepted peritoneal injection with 2% sodium pentobarbital by the standard of 0.25 ml/100 g. All rats were inverted for at least 1 min after the enema to prevent fluid outflow. We randomly single out one rat from each group to carry out model identification by the means of hematoxylin and eosin and Masson's trichrome staining. Finally, all rats received fatal dosage of anaesthetic to relieve the pain.

### 2.3. Treatments

In the HPM group, the refined mugwort floss was choosed to make moxa cones. The special herbal powder was mixed with yellow rice wine to make 90 mg herbal cake, with 0.5 cm in diameter and 0.3 cm in thickness, which contains radix aconiti lateralis preparata (10 g), cinnamon (2 g), salviae miltiorrhizae (3 g), safflower *Carthamus* (3 g), costusroot (2 g), etc. [18]. The acupoints were located at Qihai (CV6) and Tianshu (ST25, bilateral) acupoints [21]. Twice moxa cones were ignited daily by one time, a 10-day treatment. In the NC and MC groups, rats were fixed in the same manner as those in the HPM group, without any other treatment.

### 2.4. Hematoxylin and Eosin Staining

All rats were killed after treatment at the same time. Approximately 6 cm of diseased tissue from the anus was resected. One centimeter of which was washed with normal saline, soaked in 4% formaldehyde, embedded in paraffin, stained with hematoxylin and eosin, and then dehydrated in gradient alcohol and xylene. Then, the slices were observed by using the microscope (Olympus, Tokyo, Japan).

### 2.5. Histologic Fibrosis Scoring

The qualitative histologic fibrosis score was determined according to the method Theiss et al. described in [22]. The scoring criteria are given in Table 1. The degree of collagen deposition was assessed by Masson's trichrome staining kits (Nanjing Jiancheng Bioengineering Institute, Nanjing, China). About 2 sections for each rat were scored.

### 2.6. Immunohistochemistry

The samples were deparaffinized and then washed with PBS three times, repaired with citrate buffer, soaked in 0.3% hydrogen peroxide solution away from light for 20 min, and then combined with *α*-SMA (1 : 300) (Abcam, UK) and collagen III (1 : 100) (Abcam, UK). The samples were incubated with streptavidin-biotin complex (SABC) for 20 min and then colored with diaminobenzidine. The nucleus was stained with hematoxylin. Finally, the sections were covered with neutral resin. The Image-Pro software was used to evaluate the integrated optical density about the samples.

### 2.7. Western Blotting

Tissue proteins were extracted by RIPA lysis buffer (Beyotime, China) containing protease inhibitors to preserve target proteins. The concentration was detected by the BCA protein determination method (Beyotime, China). The proteins were isolated by sodium dodecyl sulfate polyacrylamide gel electrophoresis (SDS-PAGE), transferred by using the polyvinylidene fluoride (PVDF) membrane, and blocked with 5% BSA for 60 min. The membrane was incubated with primary antibodies at 4°C overnight, including *α*-SMA (1 : 1000) (Abcam, UK), collagen III (1 : 1000) (Abcam, UK), RhoA (1 : 1000) (CST, US), ROCK1 (1 : 1000) (Abcam, UK), and p-MLC (1 : 1000) (CST, US). Then, the membrane was soaked with secondary antibodies at room temperature. The membrane was automatically visualized in the gel documentation analysis system, and those images were assembled by Image Lab software.

### 2.8. Double Labeling Immunofluorescence

The samples were frozen and prepared into 10 *μ*m sections, which were immersed in PBST for 10 min, washed in PBS three times, and incubated with blocking buffer (Beyotime, China) for 90 min. Diluted primary antibodies against ROCK1 (Abcam, UK) and *α*-SMA (Abcam, UK) were soaked at 4°C overnight. Then, the secondary antibodies were added and incubated at 37°C for 1 h, and the sections were rinsed in PBS again. Finally, the sections were soaked with diluted DAPI (Beyotime, China). The fluorescence microscope was used to collect images (Olympus, Tokyo, Japan).

### 2.9. Real-Time PCR

Total RNA isolation was carried out by Trizol reagent (Invitrogen, USA) and then reverse transcribed into cDNA by a reverse transcriptase kit (Fermentas, USA). Real-time quantitative PCR analysis was performed by the method of real-time fluorescence quota PCR with the Applied Biosystems 7500 Real-Time PCR System (ABI, USA). GAPDH was used as a comparison. RNA expression was calculated relative to the housekeeping gene GAPDH. The primers used for real-time quantitative PCR analysis are given in Table 2.

### 2.10. Statistical Analysis

SPSS 24.0 was used for statistical analysis of experimental data, visualizing by GraphPad Prism 8.0. All the experimental data were expressed as the mean ± standard deviation ($\bar{X}\pm S$). If the data in each group were of normal distribution, one-way analysis of variance (ANOVA) was used for statistical analysis. When the variances were homogeneous, the pairwise comparison was showed by LSD. If not, the Games-Howell test was used. The nonparametric test was used when the data were not identical to the normal distribution. A value of *P* < 0.05 was considered significant.

## 3. Results

### 3.1. HPM Improves Histological Changes in Rats with CD-Associated Intestinal Fibrosis

**Figure 1:**
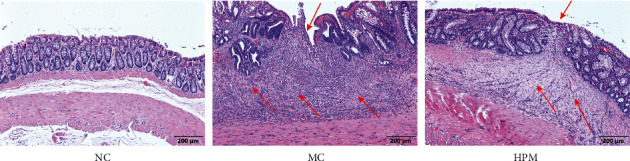
Representative histopathological changes in colon sections stained with H&E. Colons were obtained from the NC, MC, and HPM groups. Scale bar: 200 *μ*m. *n* = 8; ^*∗*^*P* < 0.05 and ^∗∗^*P* < 0.01 vs. NC; ^#^*P* < 0.05 and ^##^*P* < 0.01 vs. MC.

H&E staining showed intact mucosal epithelium and regular gland arrangement in the NC group, and no abnormal lesions appeared in the submucosa, muscular layer, or serosa layer. In the MC group, the colonic tissue was disordered, the mucosa was destroyed, and the glands had disappeared. In addition, fissured ulcers were present, fibrous tissue had proliferated around the ulcer, and a large amount of granulated tissue had formed in the mucosa, submucosa, and muscle, accompanied by abundant infiltrated fibroblasts. There are healing ulcers in the mucosal layer covered with new epithelial cells in the HPM group; some glands were irregular or had disappeared, with a small inflammatory cell infiltration and fibroblast proliferation (Figure 1).

### 3.2. Effects of HPM on the Development of Intestinal Fibrosis

**Figure 2:**
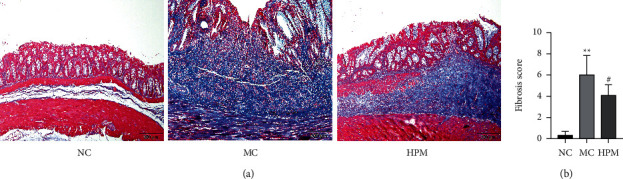
HPM decreased fibrosis scores in rats with CD-associated intestinal fibrosis. (a) Representative histologic colon sections are shown with Masson. (b) Histologic evaluation of fibrosis scores in the different groups. Scale bar: 200 *μ*m. *n* = 8; ^*∗*^*P* < 0.05 and ^∗∗^*P* < 0.01 vs. NC; ^#^*P* < 0.05 and ^##^*P* < 0.01 vs. MC.

Masson's trichrome-stained colon sections were used to determine fibrosis scores. There was normal mucosal architecture and little collagen deposition in the NC group. Rats in the MC group also exhibited obvious increases in transmural collagen deposition and histologic fibrosis scores (*P* < 0.01). However, HPM-treated rats had lower fibrosis scores than those in the MC group (*P* < 0.05), with reduced blue collagen deposition in the submucosa, mainly localized to repair tissue in the reborn epithelium (Figure 2).

**Figure 3:**
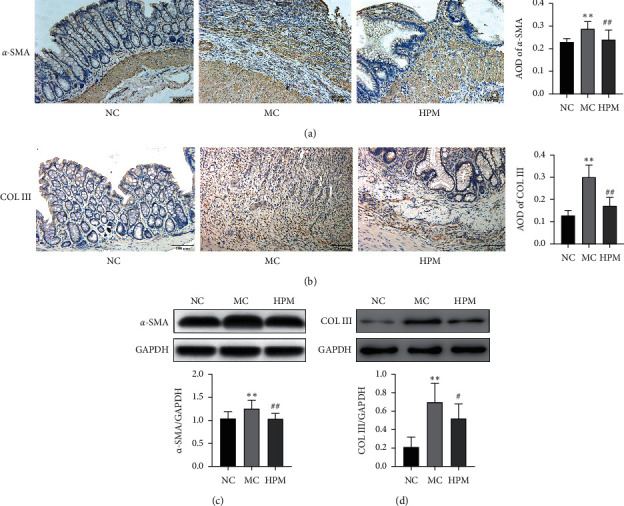
HPM regulates the expression of *α*-SMA and COL III in rats with CD-associated intestinal fibrosis. (a) Immunohistochemical staining showing *α*-SMA protein expression. (b) Immunohistochemical staining showing COL III protein expression. (c) Representative western blot band about *α*-SMA. (d) Representative western blot band about COL III. Scale bar: 100 *μ*m. *n* = 8; ^*∗*^*P* < 0.05 and ^∗∗^*P* < 0.01 vs. NC; ^#^*P* < 0.05 and ^##^*P* < 0.01 vs. MC.

As markers of fibrosis, the protein of *α*-SMA and collagen III is closely associated with the extent of intestinal fibrosis. In contrast to the NC group, immunohistochemical staining showed that the expression of *α*-SMA and collagen III was increased in the mucosa and submucosa in the MC group (*P* all ＜0.01), while HPM inhibited their expression compared with the MC group (*P* all ＜0.01) (Figures 3(a) and 3(b)). Western blot showed that the protein of *α*-SMA and collagen III in the MC group was obviously higher than the NC group (*P* all ＜0.01), while HPM decreased their expression compared with the MC group (*P* < 0.01 and *P* < 0.05) (Figures 3(c) and 3(d)).

### 3.3. Effects of HPM on the RhoA/ROCK1/MLC Pathway in CD-Associated Intestinal Fibrosis

**Figure 4:**
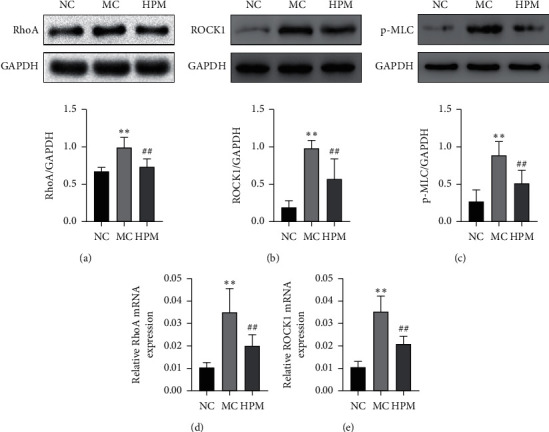
HPM regulates the RhoA/ROCK1/MLC pathway in rats with CD-associated intestinal fibrosis. (a) Representative western blot band about RhoA. (b) Representative western blot band about ROCK1. (c) Representative western blot band about p-MLC. (d) Relative mRNA expression of RhoA. (e) Relative mRNA expression of ROCK1. *n* = 8; ^*∗*^*P* < 0.05 and ^∗∗^*P* < 0.01 vs. NC; ^#^*P* < 0.05 and ^##^*P* < 0.01 vs. MC.

To assess whether the effect of HPM on CD-associated intestinal fibrosis is related to the RhoA/ROCK1/MLC pathway, real-time PCR and western blotting were applied to measure the protein of RhoA, ROCK1, and p-MLC in each group. In contrast, the mRNA and protein expression of RhoA were increased in the MC group than in the NC group (*P* all ＜0.01); compared with the MC group, HPM suppressed the protein and mRNA of RhoA (*P* < 0.01 and *P* < 0.05) (Figures 4(a) and 4(d)). Moreover, compared with the NC group, the protein and mRNA of ROCK1 were markedly increased in the MC group (*P* all  < 0.01); HPM decreased ROCK1 protein and mRNA compared to the MC group (*P* all ＜0.01) (Figures 4(b) and 4(e)). In the MC group, the expression of p-MLC was markedly increased than that in the NC group (*P* < 0.01), and HPM significantly reduced the protein expression of p-MLC in rats with CD-associated intestinal fibrosis (*P* < 0.01) (Figure 4(c)).

### 3.4. HPM Affects the Coexpression of *α*-SMA and ROCK1 in the Colon of Rats with CD-Associated Intestinal Fibrosis

**Figure 5:**
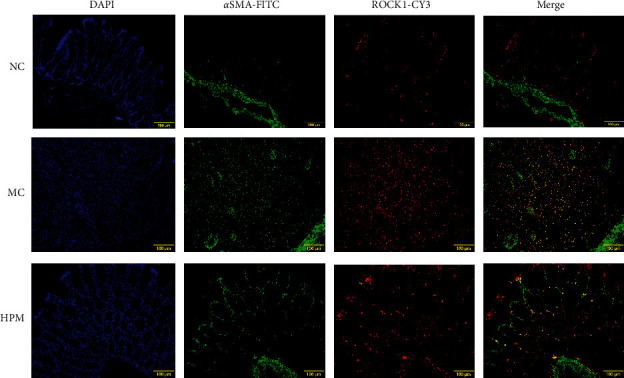
Double immunofluorescence staining of *α*-SMA (FITC, green), ROCK1 (CY3, red), and nuclei (DAPI, blue) in the colonic tissues among different groups. Bar: 100 *μ*m.

We observed increased fluorescence intensity of ROCK1 and *α*-SMA in the colonic tissues of rats with CD-associated intestinal fibrosis, and the fluorescence intensity of both ROCK1 and *α*-SMA was reduced in the HPM group. ROCK1 was mainly expressed in the mucosal and submucosal layers of the colonic tissue, especially at the site of fibrous repair in ulcers, and *α*-SMA was mainly distributed in the mucosa, submucosa, and smooth muscle layer. Compared to the NC group, there was increased coexpression of ROCK1 and *α*-SMA in model rats; the coexpression of ROCK1 and *α*-SMA in the HPM group was weaker than that in the MC group (Figure 5). So, HPM may be involved in the interaction between ROCK1 and *α*-SMA.

## 4. Discussion

The insidious onset and late clinical manifestations result in delayed diagnosis and treatment of CD-associated intestinal fibrosis. There is no established treatment for intestinal fibrosis other than mechanical intervention or surgical resection. The deposition of extracellular matrix and remodeling of cytoskeleton caused by proliferation, migration, and activation of fibroblasts are key mechanisms of intestinal fibrosis. Herb-partitioned moxibustion has been proved to inhibit collagen deposition in fibroblasts and improve intestinal fibrosis [18, 19]. Therefore, it is of great value to explore the mechanism of HPM to improve CD-associated intestinal fibrosis. In this study, we observed that the expression of RhoA, ROCK1, and p-MLC was significantly increased in the colonic tissue of CD rats with intestinal fibrosis. HPM remarkedly reduced the expression of RhoA, ROCK1, and P-MLC and improved the secretion of collagen III and *α*-SMA. Besides, the coexpression of ROCK1 and *α*-SMA proteins was observed in model rats. We hypothesize that HPM improves CD-associated intestinal fibrosis by inhibiting the RhoA/ROCK1/MLC pathway.

The results showed that the deposition of collagen and the expression of *α*-SMA and collagen III were abounding in the colon tissue of rats with CD-associated intestinal fibrosis, and HPM obviously improved collagen deposition, decreased the fibrosis score, and inhibited the levels of *α*-SMA and collagen III. Myofibroblasts are the vital cells of intestinal fibrosis, *α*-SMA is the marker of myofibroblast activation, and collagen III is one of the main collagen fibers in intestinal fibrosis. Many studies have shown that *α*-SMA and collagen III were increased in intestinal fibrosis [23–25]. As shown in our study, high levels of fibroblast proliferation were observed in the colon in CD-associated intestinal fibrosis and the collagen fibers in each layer of the intestinal wall were significantly increased; besides, collagen III and *α*-SMA were also increased, which was consistent with previous research results. In recent years, acupuncture and moxibustion therapy for CD have become increasingly popular [26, 27]. This study confirmed that HPM significantly improved collagen deposition and inhibited the expression of the two proteins. Previous studies have also shown that HPM can inhibit EMT, maintain intestinal epithelial tight junctions, and repair the damage of the enteric epithelial barrier, affecting intestinal fibrosis [18, 19, 28]. Our results confirm that HPM has an obvious effect on CD-associated intestinal fibrosis.

In this research, HPM markedly inhibited the protein of RhoA and ROCK1 in the colon of rats with intestinal fibrosis. Fibroblast migration and cytoskeletal remodeling are important to intestinal fibrosis [6]. As the member of the Ras superfamily of small guanosine triphosphate (GTP) binding proteins, RhoA is a downstream effector of certain membrane receptors. ROCK1 is a downstream signaling effector of RhoA, which is widely expressed in various tissues of noncentral systems [29]. In response to RhoA activation, ROCK1 induces a series of phosphorylation/dephosphorylation reactions, promotes the polymerization of actin cytoskeleton microfilaments, and affects cell contraction, adhesion, proliferation, and migration [30]. The RhoA/ROCK1 pathway participates in the proliferation and migration of fibroblasts, affects collagen synthesis, and is the key pathway in organ fibrosis [31–33]. It has been shown that RhoA is increased in activated primary human intestinal myofibroblasts, which are involved in the development of intestinal fibrosis [9, 10]. Moreover, the expressions of RhoA, ROCK, extracellular matrix components (collagen I–III), and *α*-SMA are increased in intestinal fibrosis [11, 34, 35]. A preclinical study also showed that the expression of ROCK was activated in the colon in CD patients with intestinal fibrosis [11]. Rho/ROCK pathway inhibitors (AMA0825, pravastatin, and Y-27632) can effectively reverse the accumulation of extracellular matrix and improve the degree of fibrosis [10, 11, 36, 37]. In this study, both RhoA and ROCK1 were increased in CD-associated intestinal fibrosis rats, which was consistent with previous studies. In addition, double immunofluorescent staining indicated the interaction between *α*-SMA and ROCK1 in CD-associated intestinal fibrosis, which suggests that the RhoA/ROCK1 pathway may be involved in the secretion of *α*-SMA by myofibroblasts in intestinal fibrosis. Existing studies on the mechanism of HPM treating intestinal fibrosis have mainly focused on TGF-*β*, ERK, and other signaling pathways; however, only a few studies have suggested that acupuncture promotes tissue repair through the RhoA/ROCK signaling pathway [38, 39]. Therefore, this study provides new evidence that HPM ameliorates CD-associated intestinal fibrosis by inhibiting RhoA and ROCK1 proteins.

Next, the study exhibited that HPM could reduce p-MLC in CD-associated intestinal fibrosis. MLC is one of the classical downstream effectors of the RhoA/ROCK1 pathway. The activation of ROCK1 induces the phosphorylation of myosin phosphatase, which increases the phosphorylation level of MLC in the cytoplasm and affects cell morphology and migration. It has been confirmed that RhoA/ROCK1 activates downstream p-MLC expression and regulates fibrosis [30, 40]. However, it is unclear whether RhoA/ROCK regulates intestinal fibrosis by activating MLC. Our results revealed that p-MLC was increased in intestinal fibrosis and HPM significantly decreased the expression of this protein.

These data preliminarily support the role of the RhoA/ROCK1/MLC pathway in rats with CD-associated intestinal fibrosis. However, RhoA/ROCK1 pathway inhibitors were not used as controls in this study, which limits the strength of the evidence. Next, we will use the specific Rho kinase inhibitor (AMA0825) and gene knockout mice for further validation studies. In addition, animal experiments are not sufficient to explain the effect of RhoA/ROCK1/MLC on intestinal fibroblasts. We will use cell culture technology to verify the effect of HPM on colonic fibroblasts through the RhoA/ROCK1/MLC pathway. Since the RhoA/ROCK pathway can also activate multiple downstream molecules, it is necessary to explore other downstream pathways associated with the Rho/ROCK pathway in the future. Although there are some limitations, our data do support our summary.

## 5. Conclusion

To sum up, this research expounded that the RhoA/ROCK1/MLC pathway was activated in rats with CD-associated intestinal fibrosis; HPM significantly reduced the level of p-MLC, RhoA, and ROCK1. Besides, HPM may reduce the interaction between ROCK1 and *α*-SMA and improved the secretion of collagen III and *α*-SMA. Therefore, these findings indicate that HPM could improve CD-associated intestinal fibrosis by inhibiting the overactivation of the RhoA/ROCK1/MLC pathway.

## Figures and Tables

**Table 1:** Criteria for histologic fibrosis scoring.

	Score	Description
Fibrosis	0	No increased collagen deposition
	1	Increased collagen deposition in the submucosa and mucosa
	2	Increased collagen deposition in the submucosa and mucosa
	3	Increased collagen deposition in the muscularis mucosa, submucosa, and mucosa; thickening and disorganization of the muscularis mucosa
	4	Increased collagen deposition in the muscularis propria, muscularis mucosa, submucosa, and mucosa
	5	Increased collagen deposition throughout all layers, including the serosa

Percent involvement	1	0–25% of the section
	2	25–50% of the section
	3	50–75% of the section
	4	75–100% of the section

**Table 2:** Primer sequences used in this study.

Gene	Forward primer sequence	Reverse primer sequence
ROCK1	5′-GGACCTTTCGGATTCAAC-3′	5′-CTGCTCACCACAACATAC-3′
RhoA	5′-GCTGGACAGGAAGATTATGAC-3′	5′-ATGATGGGCACATTTGGAC-3′
GAPDH	5-′GGAGTCTACTGGCGTCTTCAC-3′	5′-ATGAGCCCTTCCACGATGC-3′

## Data Availability

All original information of this study is contained in the article. For further details, please contact the corresponding author.
